# Phloretin Exerts Anti-Tuberculosis Activity and Suppresses Lung Inflammation

**DOI:** 10.3390/molecules22010183

**Published:** 2017-01-22

**Authors:** Dasom Jeon, Min-Cheol Jeong, Hum Nath Jnawali, Chulhee Kwak, Sungwon Ryoo, In Duk Jung, Yangmee Kim

**Affiliations:** 1 Department of Bioscience and Biotechnology, Konkuk University, Seoul 05029, Korea; dasom921012@konkuk.ac.kr (D.J.); boby8520@konkuk.ac.kr (M.-C.J.); humnath@konkuk.ac.kr (H.N.J.); chk90@konkuk.ac.kr (C.K.); 2 Korean National Tuberculosis Association, Seoul 06763, Korea; viweon@naver.com; 3 Department of Immunology, School of Medicine, Konkuk University, Seoul 05029, Korea; jungid@kku.ac.kr

**Keywords:** phloretin, natural compound, *Mycobacterium tuberculosis*, antibiotics, inflammation

## Abstract

An increase in the prevalence of the drug-resistant *Mycobacteria tuberculosis* necessitates developing new types of anti-tuberculosis drugs. Here, we found that phloretin, a naturally-occurring flavonoid, has anti-mycobacterial effects on H37Rv, multi-drug-, and extensively drug-resistant clinical isolates, with minimum inhibitory concentrations of 182 and 364 μM, respectively. Since *Mycobacteria* cause lung inflammation that contributes to tuberculosis pathogenesis, anti-inflammatory effects of phloretin in interferon-γ-stimulated MRC-5 human lung fibroblasts and lipopolysaccharide (LPS)-stimulated dendritic cells were investigated. The release of interleukin (IL)-1β, IL-12, and tumor necrosis factor (TNF)-α was inhibited by phloretin. The mRNA levels of IL-1β, IL-6, IL-12, TNF-α, and matrix metalloproteinase-1, as well as p38 mitogen-activated protein kinase and extracellular signal-regulated kinase phosphorylation, were suppressed. A mouse in vivo study of LPS-stimulated lung inflammation showed that phloretin effectively suppressed the levels of TNF-α, IL-1β, and IL-6 in lung tissue with low cytotoxicity. Phloretin was found to bind *M. tuberculosis* β-ketoacyl acyl carrier protein synthase III (mtKASIII) with high affinity (7.221 × 10^7^ M^−1^); a binding model showed hydrogen bonding of A-ring 2′-hydroxy and B-ring 4-hydroxy groups of phloretin with Asn261 and Cys122 of mtKASIII, implying that mtKASIII can be a potential target protein. Therefore, phloretin can be a useful dietary natural product with anti-tuberculosis benefits.

## 1. Introduction

**Figure 1:**
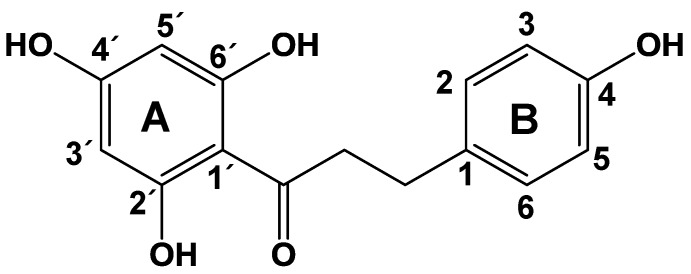
Chemical structure of phloretin.

Flavonoids are a class of polyphenolic compounds found in the human diet, mainly in fruits, vegetables, legumes, cereals, spices, nuts, and plant-derived foods, such as tea, coffee, and red wine. Dietary polyphenols exhibit a broad spectrum of pharmacological activities [1,2,3]; flavonoids are well-known for their antioxidant, anti-tumor, antimicrobial, antiviral, and anti-inflammatory activities [4]. Phloretin (3-(4-hydroxyphenyl)-1-(2,4,6-trihydroxyphenyl) propan-1-one) (Figure 1) is a dihydrochalcone with a C6–C3–C6 backbone. It is a natural compound abundant in apples (mostly in the peel) and in apple leaves, where it is present in either a free form or the glucosidic form phloridzin (phloretin 2′-*O*-glucose) [5,6,7,8]. Glycosylated derivatives of phloretin are mostly converted to the free form in the small intestine by hydrolytic enzymes [9]. Phloretin has many biological functions, including anti-oxidative and anti-cancer activities, and has been linked to the prevention of cardiovascular disease [10,11]. It was also shown to inhibit the production of inflammatory chemokines, cytokines, and differentiation factors induced by activated leukocytes in the innate immune response [12]. It was previously reported that phloretin treatment inhibits the expression of nuclear translocation of nuclear factor (NF)-κB subunit p65 and mitogen-associated protein kinase (MAPK) phosphorylation in LPS-stimulated RAW264.7 cells [13].

Tuberculosis (TB) is an infection caused by *Mycobacterium tuberculosis* that primarily affects the lung, in which it infects and activates fibroblasts, macrophages, and dendritic cells [14]. TB can be characterized by considerable inflammation and development of granulomas. TB is, therefore, a chronic inflammatory condition in which regulatory and pro-inflammatory processes occur, either mutually or stage-wise, that contribute to the establishment and progression of disease. At least one-third of the world’s population is infected with this bacterium, one of the leading infectious agents worldwide. In recent years, the control of TB has exerted pressure on chemotherapy regimens, resulting in the increasing prevalence of multidrug-resistant (MDR) and extensively drug-resistant (XDR) TB. It is, therefore, necessary to develop anti-tuberculosis drugs with enhanced activity against MDR and XDR strains, but also low toxicity, rapid mycobactericidal activity, and the ability to penetrate host cells for short-course therapy. The *Mycobacteria* cell wall, which is essential for its survival, is lipid-rich and highly impermeable, providing protection from antibiotics and allowing the bacterium to persist and to proliferate in macrophages [15]. Mycolic acids are long-chain α-alkyl-β-hydroxy fatty acids that compose up to 60% of the *Mycobacteria* cell wall and are primarily responsible for the poor permeability of the waxy cell envelope [16]. Fatty acid biosynthesis is essential for the viability of pathogenic microorganisms and has recently emerged as a target of new therapeutic agents [17,18,19]. *Mycobacteria* are unusual in that they exhibit both type I and type II fatty acid synthases [20,21]. Type I synthases are single, multifunctional polypeptides that carry out all chain elongation reactions and are typically found in yeast and mammals, whereas type II systems—in which each chain elongation reaction is carried out by a discrete enzyme encoded by a separate gene—are found in bacteria and plants [22]. β-ketoacyl-acyl carrier protein synthase III of *M. tuberculosis* (mtKASIII) links the fatty acid synthase I and II systems, catalyzing the condensation of fatty acid synthase I–derived acyl-coenzyme (Co)A with malonyl-acyl-carrier protein [23,24,25]. It is also a key condensing enzyme responsible for the initiation of type II fatty acid biosynthesis and is a potential target for novel anti-TB agents [26]. We previously reported on synthetic lead and natural compounds with broad-spectrum anti-bacterial activity as inhibitors of KAS III protein [27,28].

Though there has been great attention paid to natural products because of their beneficial effects on human health, there are no reports to date on the effects of phloretin on *M. tuberculosis*. For the first time, we investigated the anti-TB activities of phloretin. Since the anti-TB and anti-inflammatory effects gives a synergistic therapeutic effect to infection by *M. tuberculosis*, we examined the mode of action of phloretin in MRC-5 human lung fibroblasts stimulated with interferon (IFN)-γ, as well as LPS-stimulated dendritic cells. Since it has been known that dendritic cells infected by *Mycobacteria* produces large quantities of immunosuppressive cytokines, such as interleukin (IL)-1β, tumor necrosis factor (TNF)-α, and IL-6, we investigate how phloretin suppresses inflammation on LPS-stimulated dendritic cells, too [14,29]. KASIII is known as a potential target for novel anti-bacterial agents because it is an important enzyme for the initiation of type II fatty acid biosynthesis [22,23,24]. We discussed the structure-activity relationships of phoretin related to anti-TB activities and assessed the interactions between phloretin and mtKASIII, a potential target protein by molecular docking and fluorescence quenching studies. Furthermore, we investigated the in vivo effect of phloretin in a mouse model of LPS-induced lung inflammation. The results confirmed the benefits of phloretin as a dietary natural compound for the suppression of TB and associated lung inflammation.

## 2. Results and Discussion

### 2.1. Anti-TB Activities of Phloretin

**Figure 2:**
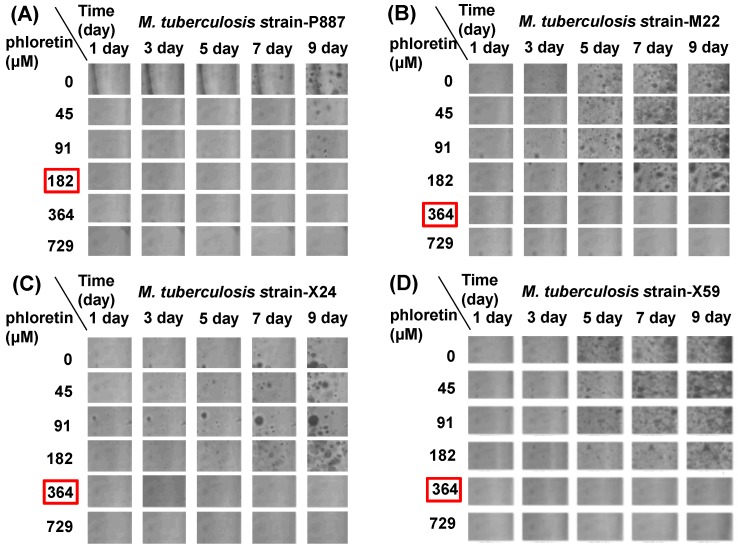
Effects of phloretin on the growth of (**A**) *M. tuberculosis* P887 (H37Rv); (**B**) *M. tuberculosis* M22 (multidrug resistant); (**C**) *M. tuberculosis* X24 (extensively drug resistant); and (**D**) *M. tuberculosis* X59 (extensively drug resistant) strains. Red squares denote the MIC_90_ for each strain.

Phloretin is a natural food product, present in apple and strawberries that may improve acute and chronic disease [11]. Apples are known to inhibit colon, lung, and prostate cancers [30], to improve lung function, and to decrease the risk of respiratory diseases, such as chronic obstructive pulmonary disease [31]. In the present study, we investigated for the first time that phloretin has anti-mycobacterial activities against *M. tuberculosis* strain H37Rv and MDR and XDR clinical isolates, and also suppresses lung inflammation. Phloretin inhibited the growth of strain H37Rv, with a MIC_90_ (lowest concentration to produce 90% inhibition of *M. tuberculosis* growth) of 182 μM, and MDR (M22) and XDR (X24 and X59) isolates at MIC_90_ = 364 μM (Figure 2A–D). Therefore, phloretin may potentially be taken with conventional anti-TB drugs to protect against TB. Recently, we demonstrated that the natural flavonoid isorhamnetin, which is abundant in apples, cherries, and blackberries, showed anti-TB activity against *M. tuberculosis* H37Rv, multi-drug- and extensively drug-resistant clinical isolates, too [32]. Therefore, natural flavonoids may be a good source of anti-TB agents.

### 2.2. Effects of Phloretin on mRNA Expression of Inflammatory Cytokines in IFN-γ-Stimulated MRC-5 Cells and in LPS-Stimulated Dendritic Cells

IFN-γ is a cytokine produced by activated T cells and natural killer cells; it is one of the major cytokines responsible for activating macrophages and induces inflammation via nitric oxide synthase, cyclooxygenase-2, IL-1β, and IL-12 upregulation and activation [33,34]. *M. tuberculosis* infection occurs primarily in the lungs and lymph nodes and is characterized by the formation of granulomas [35], which are organized immune cell structures consisting of macrophages, fibroblasts, neutrophils, and lymphocytes [36,37]. Fibroblasts in connective tissue can be infected by and support the replication of *M. tuberculosis* [38], which can interact with cells of the innate and adaptive immune systems, inducing the secretion of chemokines and cytokines, including IL-1β, IL-6, IFN-γ, IL-12, and TNF-α.

**Figure 3:**
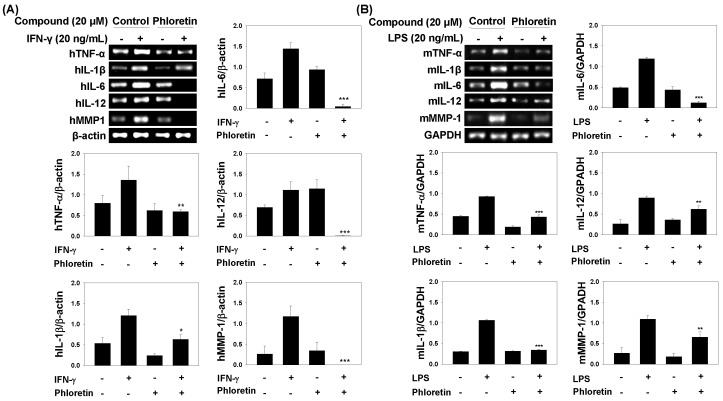
Effects of phloretin on mRNA expression of inflammatory cytokines (**A**) tumor necrosis factor (TNF)-α, interleukin (IL)-1β, IL-6, IL-12, matrix metalloproteinase (MMP)-1, and β-actin in interferon (IFN)-γ-treated MRC-5 cells and their quantitative analysis of reverse transcription polymerase chain reaction results; and (**B**) TNF-α, IL-1β, IL-6, IL-12, MMP-1, and Glyceraldehyde 3-phosphate dehydrogenase (GAPDH) in LPS-treated dendritic cells and their quantitative analysis of reverse transcription polymerase chain reaction results. Cells were untreated (negative control) or stimulated with 20 ng/mL IFN-γ for MRC-5 cells and 20 ng/mL LPS for dendritic cells in the presence or absence of phloretin for 3 h. Relative expression was quantified using ImageJ, and bars represent mean expression level ± standard deviation relative to β-actin for IFN-γ-treated MRC-5 cells and GAPDH for LPS-treated dendritic cells. * *p* < 0.05, ** *p* < 0.005, *** *p* < 0.001 vs. cells treated with IFN-γ or LPS only.

To clarify the mechanism by which phloretin suppresses inflammation induced by IFN-γ on human lung fibroblast MRC-5 cells, we examined the mRNA expression of factors downstream of IFN-γ—including the inflammatory cytokines IL-1β, IL-6, IL-12, and TNF-α, as well as matrix metalloproteinase (MMP)-1—by reverse transcription-polymerase chain reaction (RT-PCR). IFN-γ induced an upregulation of these factors after 3 h, whereas pretreatment with phloretin decreased the levels of TNF-α, IL-1β, IL-6, IL-12, and MMP-1 by 55%, 48%, 97%, 100%, and 100%, respectively, as compared to the levels in untreated cells (Figure 3A).

*M. tuberculosis* infection also activates dentritic cells and increases production of cytokines, such as TNF-α, IL-1β, IL-6, and IL-12p70 [14,29]. Therefore, we examined the mRNA expression of the inflammatory cytokines IL-1β, IL-6, IL-12, and TNF-α, and MMP-1 in LPS-stimulated dendritic cells by RT-PCR. Pretreatment with phloretin decreased the levels of TNF-α, IL-1β, IL-6, IL-12, and MMP-1 by 53%, 68%, 90%, 30%, and 40%, respectively, as compared to the levels in untreated cells (Figure 3B).

### 2.3. Effects of phloretin on IFN-γ-Induced Protein Expression

**Figure 4:**
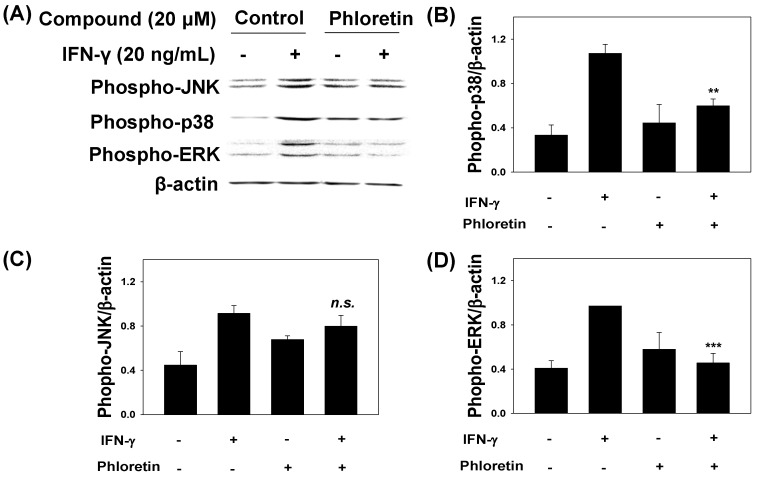
JNK, p38 MAPK, and ERK expression in MRC-5 cells pretreated with phloretin and stimulated with interferon (IFN)-γ. (**A**) MAPK levels were evaluated by Western blot, with β-actin used as a loading control; (**B**–**D**) relative intensities of phosphorylated JNK, p38 MAPK, and ERK normalized to β-actin, respectively. Data represent mean ± standard deviation. ** *p* < 0.005, *** *p* < 0.001 vs. cells treated with IFN-γ only.

In order to understand the signaling pathways activated by *Mycobacteria*, we investigated the effects of phloretin on the phosphorylation status of c-Jun *N*-terminal kinase (JNK), p38 MAPK, and extracellular signal-regulated kinase (ERK) in MRC-5 cells stimulated with IFN-γ. Phloretin reduced the phosphorylation of JNK, p38 MAPK, and ERK to 13%, 44%, and 53%, respectively (Figure 4A–D).

### 2.4. Inflammatory Cytokine Levels in INF-γ-Stimulated MRC-5 Cells and LPS-Stimulated Dendritic Cells

**Figure 5:**
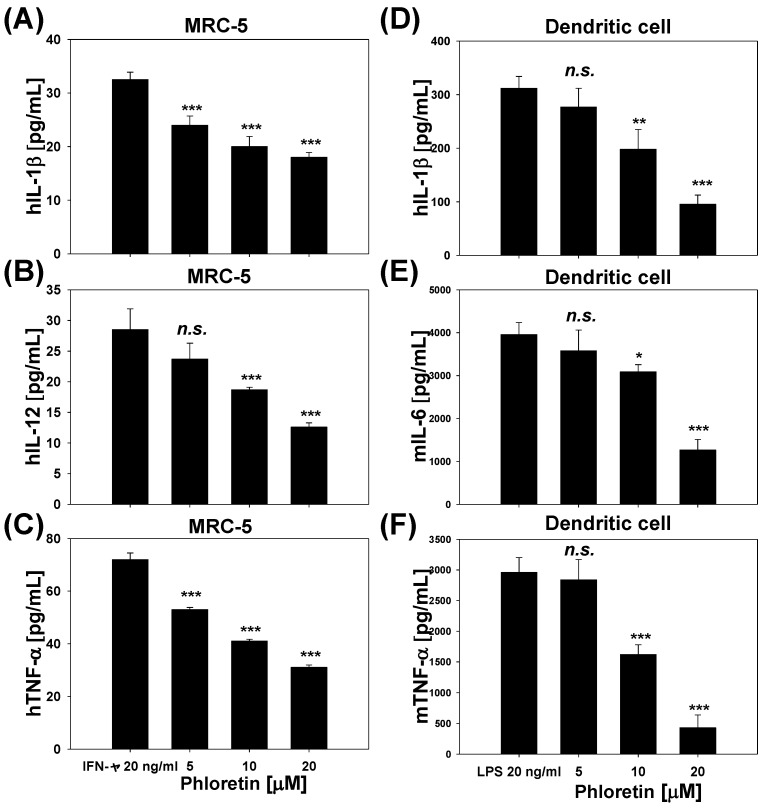
Levels of (**A**) interleukin (IL)-1β; (**B**) IL-12; and (**C**) tumor necrosis factor (TNF)-α in the culture medium of interferon (INF)-γ-stimulated MRC-5 cells; and (**D**) IL-1β; (**E**) IL-6; and (**F**) tumor necrosis factor (TNF)-α in the culture medium of lipopolysaccharide-stimulated dendritic cells treated with indicated concentrations of phloretin for 24 h. Data represent mean ± standard deviation of three independent experiments. * *p* < 0.05, ** *p* < 0.005, *** *p* < 0.001 vs. cells treated with IFN-γ only (**A**–**C**) or LPS only (**D**–**F**).

We also examined the effects of phloretin on TNF-α, IL-1β, and IL-12 secretion in MRC-5 cells stimulated with 20 ng/mL INF-γ (Figure 5A–C). At 10 and 20 μM concentrations, phloretin suppressed IL-1β production by 39% and 45% respectively (Figure 5A), IL-12 levels by 35% and 56%, respectively (Figure 5B), and TNF-α production by 43% and 57% respectively (Figure 5C), as compared to cells that did not receive phloretin treatment. We then measured the effects of phloretin on TNF-α, IL-1β, and IL-6 secretion in dendritic cells stimulated with 20 ng/mL LPS (Figure 5D–F). At 10 and 20 μM, phloretin suppressed IL-1β production by 37% and 69%, respectively (Figure 5D); IL-6 levels by 22% and 68%, respectively (Figure 5E); and TNF-α production by 45% and 85%, respectively (Figure 5F), as compared to cells without phloretin treatment.

### 2.5. TNF-α, IL-1β, and IL-6 Levels in LPS-Stimulated Mouse Lung Tissue

**Figure 6:**
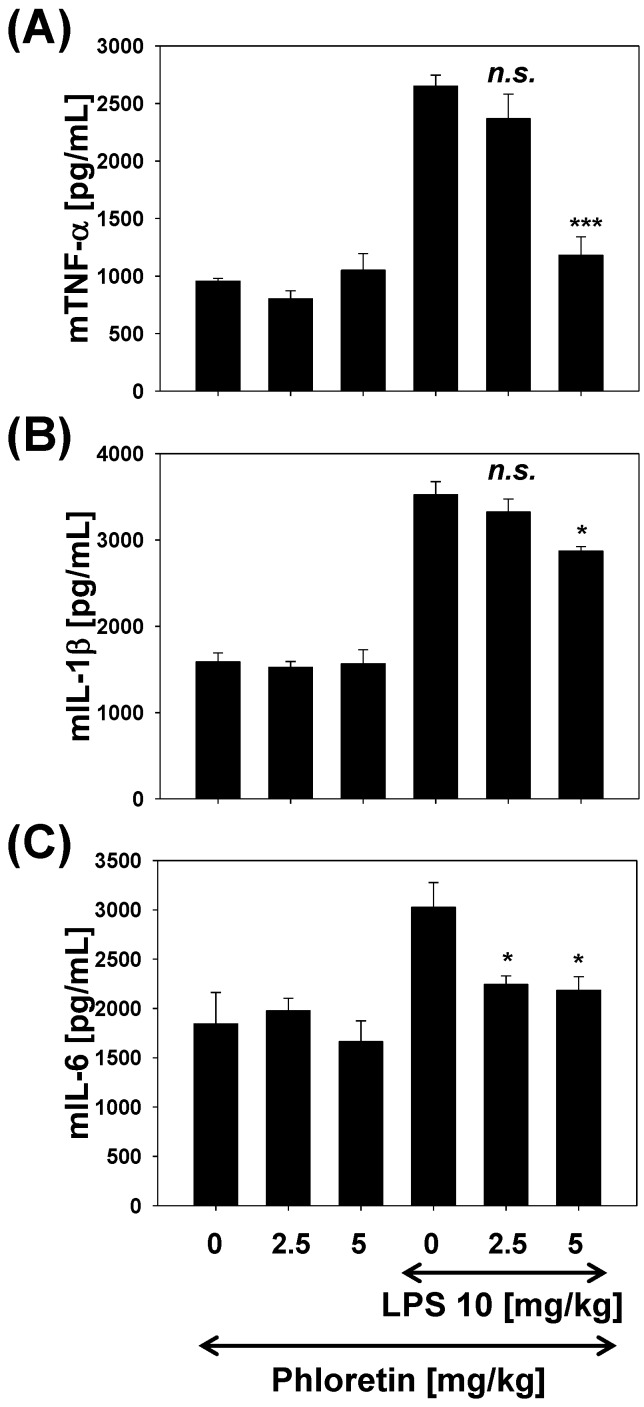
Inhibition of the pro-inflammatory cytokines (**A**) tumor necrosis factor (TNF)-α; (**B**) interleukin (IL)-1β; and (**C**) IL-6, in a mouse model of lipopolysaccharide (LPS)-stimulated lung inflammation. * *p* < 0.05, *** *p* < 0.001 vs. cells treated with LPS only.

In order to demonstrate the potency of phloretin as an anti-inflammatory agent in lung inflammation, we investigated the effects of phloretin on inflammation in vivo. We measured the effect of phloretin on the production of pro-inflammatory cytokines TNF-α, IL-1β, and IL-6 in lung homogenates from 10 mg/kg LPS-stimulated mice (Figure 6). At 2.5 and 5 mg/kg, phloretin inhibited TNF-α production in vivo by 11% and 56%, respectively (Figure 6A); IL-1β by 6% and 19%, respectively (Figure 6B); and IL-6 by 26% and 28% (Figure 6C), respectively.

### 2.6. Cytotoxicity of Phloretin in Mammalian Cells and in LPS-Stimulated Mice

**Figure 7:**
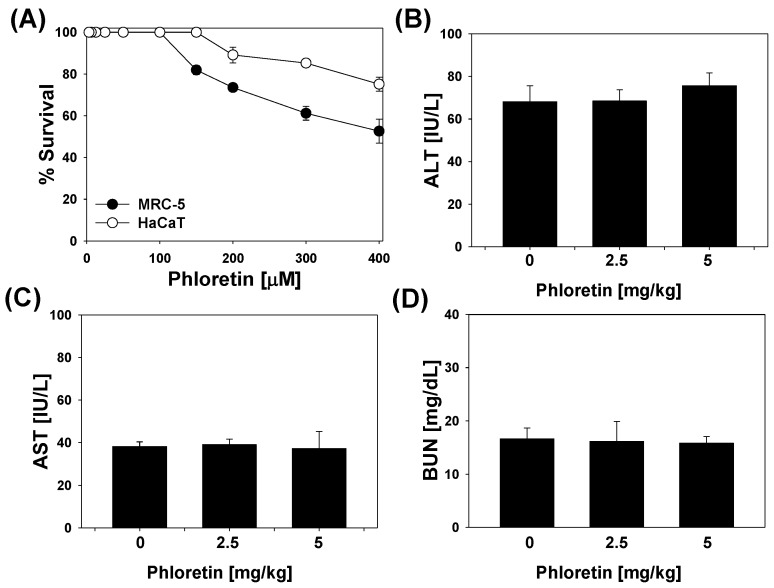
(**A**) Cell viability upon treatment with phloretin. MRC-5 human lung fibroblasts and HaCaT non-cancerous human keratinocytes were treated with phloretin for 24 h and viability was assessed with the 3-(4,5-dimethylthiazol-2-yl)2,5-diphenyl tetrazolium bromide (MTT) assay; the effects of phloretin on serum levels of (**B**) alanine transaminase (ALT); (**C**) aspartate aminotransferase (AST); and (**D**) blood urea nitrogen (BUN). Data represent mean ± standard deviation.

We examined the toxicity of phoretin in mammalian cells (MRC-5 and HaCaT cells) with the 3-(4,5-dimethylthiazol-2-yl)2,5-diphenyl tetrazolium bromide (MTT) assay. Phloretin concentrations of up to 100 μM did not affect the viability of MRC-5 cells (Figure 7A); even at concentrations of 400 μM, viability was >50% in MRC-5 cells. On the other hand, the survival of HaCaT non-cancerous human keratinocytes was unaffected up to 150 μM; viability was >75%, even at concentrations of up to 400 μM. At its MIC_90_ (182 μM) against M. tuberculosis-P887 strain, survival rates of HaCaT cells and MRC-5 cells were 93% and 78%, respectively, while at its MIC_90_ (364 μM) against MDR and XDR strains, cell survival rates were 76% and 55%, respectively.

We also investigated the in vivo toxicity of phloretin by measuring serum alanine transaminase, aspartate aminotransferase, and blood urea nitrogen levels. We found that the levels of these blood parameters in mice treated with 2.5 or 5 mg/kg phloretin were not significantly different from those in mice treated with LPS only (Figure 7B–D). In addition, the phloretin-treated mice did not show visible signs of toxicity, such as weight loss, twisting, irritability, or death.

### 2.7. Phloretin–mtKASIII Binding Affinity and Docking Study of Phloretin with mtKASIII

**Figure 8:**
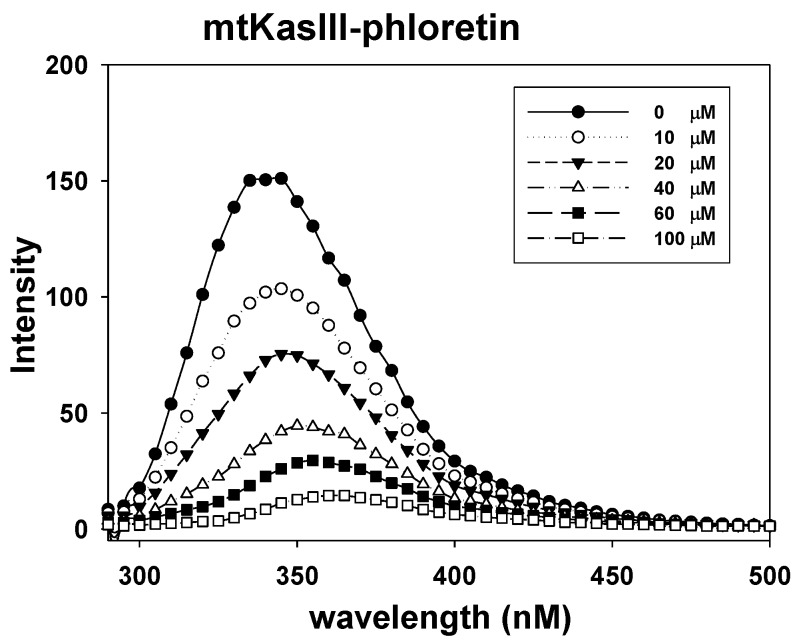
Fluorescence spectra of mtKASIII in the presence of 0, 10, 20, 40, 60, and 100 μM phloretin at pH 7.0. Samples were excited at 290 nm, and emission spectra were recorded at 290–600 nm.

In order to find the possible target protein for anti-TB activity of phloretin, we investigated the interaction between phloretin and mtKASIII. mtKASIII is a key condensing enzyme responsible for the initiation of type II fatty acid biosynthesis and can be a potential target for novel anti-TB agents. The fluorescence titration curves for mtKASIII with phloretin indicated that the tryptophan fluorescence of mtKASIII was significantly quenched in the presence of phloretin (Figure 8). Phloretin bound tightly to mtKASIII with a binding affinity of 7.221 × 107 M^−1^.

**Figure 9:**
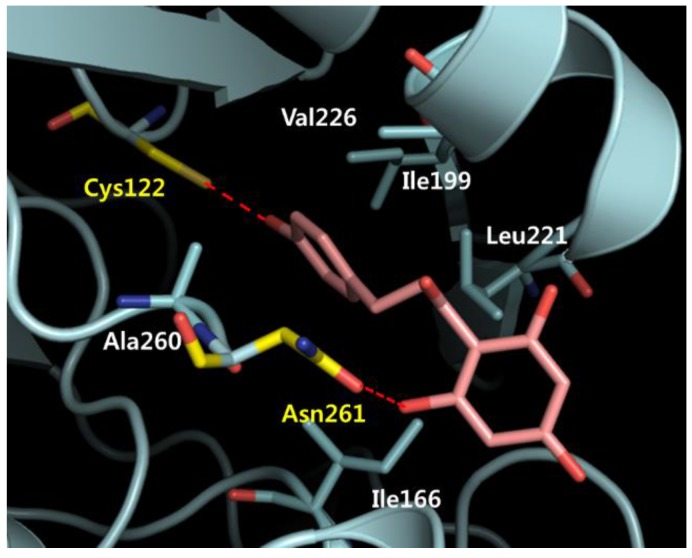
Binding of phloretin and mtKASIII. Hydrophobic residues and those participating in hydrogen bonding are shown in white and yellow, respectively.

A docking analysis revealed hydrogen bonding networks between the Asn261 of mtKASIII and the 2′-hydroxy groups of the A-ring of phloretin and between the Cys122 of mtKASIII and the 4′-hydroxy groups of the B-ring of phloretin (Figure 9). The B-ring of phloretin formed additional hydrophobic interactions with Ile166, Ile199, Leu221, Val226, and Ala260 of mtKAS III. Our current results indicate that phloretin exhibited strong binding to mtKASIII, mainly via hydrogen bonding and hydrophobic interactions, which may contribute to the inhibition of initial type II fatty acid elongation step by mtKASIII in *M. tuberculosis*. These results confirmed that mtKASIII can be a potential target for novel anti-TB agents.

## 3. Materials and Methods

### 3.1. Phloretin

Phloretin was purchased from Sigma-Aldrich (St. Louis, MO, USA) and the purity was determined to be 99% by high-performance liquid chromatography. The chemical was dissolved in dimethylsulfoxide to obtain a 10 mg/mL stock solution.

### 3.2. Evaluation of Anti-TB Activity

The anti-tuberculosis activity of phloretin against MDR and XDR M. tuberculosis H37Rv strains was determined as previously described [32]. Briefly, the culture broth (166 µL) of *M. tuberculosis* H37Rv, MDR, and XDR clinical isolates was combined at 37 °C with 500 µL of 0.5% liquid agarose. A 10 µL volume of this mixture was loaded into an inlet well to form microfluidic agarose channels. The bacteria were immobilized when the gel solidified at room temperature. Phloretin (0–729 µM), the positive control isoniazid (0–16 µM, Sigma-Aldrich), and Middlebrook 7H9 broth containing 10% oleic albumin dextrose catalase were loaded onto the gel; the compounds and culture medium diffused towards the microfluidic agarose channels. The 96-well microfluidic agarose channel chip was incubated at 37 °C and the boundary area between the liquid medium loading well and microfluidic agarose channel was imaged at 1, 3, 5, 7, and 9 days on an inverted microscope using a 40× objective lens. Bacterial growth was determined from the images. The dark grey spots corresponding to *M. tuberculosis* strains in time-lapse images were converted to digital information by image processing. Susceptibility to the agent was determined by evaluating the area of *M. tuberculosis* coverage and calculating the lowest concentration inhibiting *M. tuberculosis* growth by 90% (MIC_90_). All assays were performed at least three times.

### 3.3. Reverse Transcription-Polymerase Chain Reaction

Human lung fibroblast MRC-5 cells were purchased from the American Type Culture Collection (Manassas, VA, USA). Human lung fibroblast MRC-5 cells were stimulated with or without 20 ng/mL IFN-γ in the presence or absence of phloretin for 3 h. Dendritic cells were stimulated with or without 20 ng/mL LPS in the presence or absence of phloretin for 3 h. The mRNA expression of IL-1β, IL-6, hIL-12, TNF-α, MMP-1, β-actin, and GAPDH (Glyceraldehyde 3-phosphate dehydrogenase) was evaluated by RT-PCR using previously-reported primers [32]. Signal intensity was quantified by densitometry using ImageJ software (National Institutes of Health, Bethesda, MD, USA) [39].

### 3.4. Western Blot

Equal amounts of protein (20 μg) isolated from MRC-5 cells were electrophoretically separated on 10% sodium dodecyl sulfate polyacrylamide gels and then transferred to polyvinylidene difluoride membranes [32] that were blocked and incubated with primary antibodies against phosphorylated JNK, phosphorylated ERK, phosphorylated p38, and β-actin at 4 °C overnight [40]. After washing three times with tris-buffered saline containing Tween 20, the membranes were incubated with horseradish peroxidase-conjugated secondary antibodies at room temperature for 4 h, followed by incubation with Luminol/Enhancer solution. The relative density of protein bands was quantified using ImageJ software.

### 3.5. Enzyme-Linked Immunosorbent Assay

The inhibitory effect of phloretin on inflammatory cytokine production was evaluated in MRC-5 human lung fibroblasts as well as LPS-stimulated dendritic cells by enzyme-linked immunosorbent assay (ELISA). Inflammatory cytokine levels were calculated based on a standard curve using recombinant cytokines provided in the ELISA kits as previously described [41]. Values are reported as the mean ± standard deviation of at least three independent experiments.

### 3.6. In Vivo Mouse Model of LPS-Stimulated Lung Inflammation

BALB/c mice where phloretin exerts anti-tuberculosis activity and suppresses lung inflammation assigned to six experimental groups, with three mice in each group. Samples were prepared in phosphate-buffered saline. Mice were pre-administered phloretin (2.5 or 5 mg/kg) by intraperitoneal injection. After 1 h, the mice received another intraperitoneal injection with 10 mg/kg LPS and were retained for 12 h before sacrifice. After centrifuging the lung homogenates at 13,000 rpm for 15 min at 4 °C, the supernatants were measured by ELISA as described previously [32]. Serum alanine transaminase and aspartate aminotransferase were analyzed using the Reitman-Frankel method as follows: The substrate reagent and serum were co-incubated for 60 min at 37 °C. Then color reagent was added and the mixture was further incubated for 20 min at room temperature. After that, 0.4 N NaOH was added and the absorbance was monitored at 505 nm. Blood urea nitrogen levels were measured using a urease-indophenol method kit (Asan pharmaceutical, Hwaseong, Korea) as follows: the enzyme solution and the serum were co-incubated for 5 min at 37 °C. Then color reagent was added and the mixture was incubated for 10 min at 37 °C. The absorbance was measured at 580 nm. All experiments and protocols for animal care were reviewed and approved by the Institutional Animal Care and Use Committee of Konkuk University (KU15046).

### 3.7. Cytotoxicity of Phloretin against Mammalian Cells

MRC-5 and HaCaT human keratinocytes were purchased from the American Type Culture Collection (Manassas, VA, USA). The 3-(4,5-dimethylthiazol-2-yl)2,5-diphenyl tetrazolium bromide (MTT) assay was used to assess the viability of MRC-5 and HaCaT cells exposed to phloretin (0–400 μM). Cell viability was measured at 570 nm with a microplate reader and expressed as percentage values representing the mean of at least three independent experiments.

### 3.8. Construction, Expression, and Purification of mtKASIII

The *fabH* gene encoding mtKASIII amplified from *M. tuberculosis* (H37Rv) genomic DNA was obtained from The Korean Institute of Tuberculosis (Seoul, Korea). The forward and reverse primers 5′-TAG GAC GCA TAT GAC GGA GAT CGC CAC-3′ and 5′-CTG GCT GGA TCC GAT CTT CGC GCG CTC A-3′ with *Nde*I and *Bam*HI restriction sites, respectively, were used to amplify mtKASIII. Purified PCR products were first cloned into the pGEM-T-Easy vector (Promega, Madison, WI, USA) and mtKASIII fragments were inserted into pET15b via *Nde*I and *Bam*HI sites to obtain pET-15b/mtKASIII, which was transformed into *Escherichia coli* BL21 (DE3) cells. Expression and purification of mtKASIII were carried out as described previously for *Staphylococcus aureus* KASIII protein [27].

### 3.9. Fluorescence Quenching between mtKASIII Protein and Phloretin

The binding constants of phloretin and mtKASIII protein were determined at 25 °C with an RF-5301PC spectrofluorophotometer (Shimadzu, Kyoto, Japan). Phloretin was titrated with 10 μM mtKASIII protein solution in sodium phosphate buffer (50 mM) containing NaCl (100 mM) at pH 8.0, with a protein-to-inhibitor ratio of 1:10. The samples were placed in a 2 mL cuvette and absorbance was measured with excitation and emission path lengths of 10 nm. Fluorescence quantum yields of mtKASIII in the presence of increasing concentrations of phloretin were determined by measuring tryptophan emission. Detailed procedures are described in our previous report [42,43].

### 3.10. Docking Studies

The X-ray crystallographic structure of free form mtKASIII (1M1M.pdb) was used to evaluate its binding with phloretin [44]. We defined the active site and considered receptor-substrate interactions. Computations were performed on a Linux platform using DS modeling/CDOCKER (Biovia, San Diego, CA, USA) and a CHARMM force field [43]. The algorithm assumed a rigid protein and permitted ligand flexibility. The final docking poses were refined by a minimization step. The results were reported as an energy term that included both protein-ligand interaction and internal ligand strain. A higher energy value indicates more favorable binding.

### 3.11. Statistical Analyses

At least three independent samples were included in each analysis. Data were analyzed using GraphPad InStat v.3.05 software (GraphPad Inc., La Jolla, CA, USA). Values were considered statistically significant at *p* < 0.05.

## 4. Conclusions

*Mycobacteria* have a lipid-rich, highly impermeable cell wall that provides protection from most antibiotics. Phloretin is one of the natural occurring chalcones (1,3-diaryl-2-propen-1-ones) and has a flexible structure, which is presumed to enhance its membrane permeability. Some synthetic chalcones and their synthetic derivatives have demonstrated inhibitory activity against *M. tuberculosis* strain H37Rv in previous studies [45,46,47,48,49].

As shown in the binding model, phloretin can go deep into the active site of mtKASIII, resulting in hydrogen bonding with catalytic residue Cys122, as well as Asn261. This may cause the inhibition of mtKASIII, resulting in anti-tubercular activities of phloretin. The mechanism could be investigated by protein assays in future research. The *Mycobacteria* cell wall, which is essential for its survival, is lipid-rich and highly impermeable, providing protection from antibiotics and allowing the bacterium to persist and to proliferate in macrophages. From our current results, the flexible, open-chain structure of phloretin is the key factor for the membrane permeability against the *Mycobacteria* cell wall.

In this study, we showed that treatment with phloretin decreased IL-1β, IL-6, IL-12, TNF-α, and MMP-1 mRNA levels in IFN-γ-stimulated human lung fibroblast MRC-5 cells, as well as LPS-stimulated dendritic cells. *Mycobacteria* activate signaling pathways involved in inflammation, including MAPK and NF-κB [50], and *M. tuberculosis* has been shown to induce MMP-1 expression in a signal transducer and activator of transcription 3/p38 MAPK/NF-κB-dependent manner, thereby stimulating collagen cleavage in human lung fibroblast MRC-5 cells [51]; these effects are attenuated by phloretin. The current data suggest that phloretin modulates the expression of pro-inflammatory cytokines via inhibition of p38 MAPK/ERK signaling. Phloretin may target ERK and p38 MAPK signaling downstream of IFN-γ. In vitro and in vivo blood tests in LPS-stimulated mouse models indicate that phloretin has minimal toxicity. By phloretin, the levels of the pro-inflammatory cytokines TNF-α, IL-1β, and IL-6 were suppressed in lung homogenates from mice with LPS injection in vivo.

Though the MIC_90_ of phloretin is quite high for clinical purposes foods, such as apple, can be a promising dietary source of phloretin as an anti-TB molecule. Our in vivo and in vitro results showed that phloretin may be a safe and effective natural compound for the prevention of TB, as well as lung inflammation.
